# Developing a harmonized heat warning and information system for Ontario: a case study in collaboration

**DOI:** 10.17269/s41997-020-00337-y

**Published:** 2020-06-10

**Authors:** Dave Henderson, Louise Aubin, Kevin Behan, Hong Chen, Helen Doyle, Stephanie Gower, Melissa MacDonald, Carol Mee, Gregory R. A. Richardson, Greg Rochon, Mira Shnabel, Jay Storfer, Abderrahmane Yagouti, Anna Yusa

**Affiliations:** 1 Health and Air Quality Forecast Services, Meteorological Service of Canada, Environment and Climate Change Canada (Government of Canada), *At time of writing, Ottawa, ON Canada; 2 Health Protection, Public Health, Health Services, Region of Peel, Mississauga, ON Canada; 3 Clean Air Partnership, Toronto, ON Canada; 4 Environmental and Occupational Health, Public Health Ontario, Toronto, ON Canada; 5 Health Protection Division, Community and Health Services Department, York Region Public Health, *At time of writing, Newmarket, ON Canada; 6 Strategy and Preventive Health, Toronto Public Health, Toronto, ON Canada; 7 Health and Air Quality Forecast Services, Meteorological Service of Canada, Environment and Climate Change Canada (Government of Canada), Dartmouth, NS Canada; 8 Healthy Public Policy, Toronto Public Health, *At time of writing, Toronto, ON Canada; 9 Climate Change and Innovation Bureau, Health Canada (Government of Canada), RM 9-076 - 269 Laurier Ave West, Ottawa, ON K1A 0K9 Canada; 10 Emergency Management, North Bay Parry Sound District Health Unit, North Bay, ON Canada; 11 Health Protection Division, Community and Health Services Department, York Region Public Health, Newmarket, ON Canada; 12 Renewable Energy and Electricity Division, Low Carbon Energy Sector, Natural Resources Canada (Government of Canada), Ottawa, ON Canada; 13 Centre for Climate Services, Environment and Climate Change Canada (Government of Canada), Gatineau, QC Canada; 14 Climate Change and Innovation Bureau, Health Canada (Government of Canada), *At time of writing, Toronto, ON Canada

**Keywords:** Extreme heat, Collaboration, Public health intervention, Weather warning system, Chaleur extrême, Collaboration, Intervention en santé publique, Système d’avertissement météorologique

## Abstract

**Background:**

Heat wave early warning systems help alert decision-makers and the public to prepare for hot weather and implement preventive actions to protect health. Prior to harmonization, public health units across Ontario either used independent systems with varying methodologies for triggering and issuing public heat warnings or did not use any system. The federal government also issued heat warnings based on different criteria. During heat events, adjacent public health units in Ontario and the federal government would routinely call heat warnings at different times with separate public messages, leading to confusion. This article describes the collaborative process and key steps in developing a harmonized Heat Warning and Information System (HWIS) for Ontario.

**Setting:**

Public health units across Ontario, Canada, collaborated with the federal and provincial government to develop the harmonized HWIS for Ontario.

**Intervention:**

In 2011, stakeholders identified the need to develop a harmonized system across Ontario to improve heat warning services, warning criteria, and health messaging. Through a 5-year process facilitated by a non-governmental organization, the three levels of government collaborated to establish the Ontario HWIS.

**Outcomes:**

The province-wide HWIS was implemented in 2016 with the Ontario Ministry of Health and Long-Term Care’s release of the harmonized HWIS Standard Operating Practice, which outlined the notification and warning process.

**Implications:**

The lessons learned could help spur action in other provinces and jurisdictions internationally in the development of similar health evidence-based warning systems, including in particular those for protecting public health during extreme heat events.

**Electronic supplementary material:**

The online version of this article (10.17269/s41997-020-00337-y) contains supplementary material, which is available to authorized users.

## Résumé

**Contexte:**

Les systèmes d’alerte rapide de vague de chaleur aident les décideurs et le public à se préparer au temps chaud et à prendre des mesures de protection sanitaire préventives. Avant l’harmonisation, les services de santé publique de l’Ontario utilisaient des systèmes indépendants qui faisaient appel à diverses méthodes pour déclencher et diffuser des avertissements de chaleur. Dans certains services, aucun système n’était en place. Le gouvernement fédéral émettait également des avertissements de chaleur en fonction de différents critères. Pendant les périodes de chaleur, les bureaux de santé publique adjacents de l’Ontario ainsi que le gouvernement fédéral émettaient régulièrement des avertissements à des moments et au contenu différents, ce qui créait de la confusion. Le présent article décrit le processus de collaboration qui a mené à l’élaboration d’un système d’avertissement et d’information de chaleur (SAIC) harmonisé pour l’Ontario et les étapes clés de ce processus.

**Lieu:**

Les bureaux de santé publique de l’ensemble de l’Ontario, au Canada, ont collaboré avec les gouvernements fédéral et provincial à l’élaboration du SAIC harmonisée pour l’Ontario.

**Intervention:**

En 2011, des intervenants ont cerné le besoin d’élaborer un système harmonisé à l’échelle de l’Ontario pour améliorer les services d’avertissement de chaleur, les critères d’émission des avertissements et les messages sur la santé. Dans le cadre d’un processus de cinq ans dirigé par une organisation non gouvernementale, les trois ordres d’administration ont collaboré à la mise sur pied du SAIC de l’Ontario.

**Résultats:**

Le SAIC a été lancé à l’échelle de la province en 2016. À cette occasion, le ministère de la Santé et des Soins de longue durée de l’Ontario a publié une norme de pratique pour le SAIC harmonisé, qui décrit le processus d’avis et d’avertissement.

**Implications:**

Les leçons apprises pourraient contribuer à inciter d’autres provinces et des administrations ailleurs dans le monde à mettre au point des systèmes d’avertissement semblables fondés sur des données médicales probantes, en particulier lorsqu’il s’agit de protéger la santé publique en périodes de chaleur extrême.

## Introduction

Heat wave early warning systems alert the public and health officials to impending hot weather and activate interventions to protect the public from the negative health impacts from extreme heat. Beginning in 2012, public health units (PHUs) across Ontario worked with various partners to develop a province-wide Heat Warning and Information System (HWIS). This was a unique collaboration that brought together all orders of government with different mandates, needs, and degrees of activity and experience around heat warning systems. All partners involved came together voluntarily and agreed on a common purpose and process. Unlike other activities spurred by heat emergencies, the development of a HWIS for Ontario was undertaken proactively and driven by public health units. This case study outlines the process for establishing the harmonized system in Ontario, including the impetus for action, the role of key partners, and the launch of the new system. Several key lessons are described that could help other jurisdictions looking to implement their own harmonized systems.

## Context

Heat events can have a major impact on health. In the summer of 2003, Europe experienced unseasonably hot weather which resulted in approximately 70,000 deaths (Robine et al. 2008). Canada is not exempt from heat-related deaths. In 2009, an extreme heat event contributed to 156 excess deaths in the province of British Columbia (Kosatsky 2010), while in Quebec, extreme heat events led to more than 280 excess deaths in 2010 (Bustinza et al. 2013) and an estimated 86 deaths in 2018 (Lebel et al. 2019).

High temperatures in summer have been associated with increases in mortality across the province of Ontario (Chen et al. 2016). In the City of Toronto, for example, Pengelly et al. (2007) estimated heat contributed to an average of 120 deaths per year. The research shows that heat is most likely to affect already vulnerable populations such as people with low income, those who are very young or old, or those who experience homelessness (Berry and Richardson 2016). Climate change projections indicate the number of hot days in Ontario could double by mid-century and triple by the end of the century (Casati et al. 2013).

Heat-related health risks can be reduced through systematic development of heatwave early warning systems (WHO and WMO 2015). These systems, which include the Heat Warning and Information System (HWIS) described in this article, alert the public and decision-makers to prepare for hot weather and implement measures to avoid negative health effects (Lowe et al. 2011). Post-event analyses of extreme heat events in Canada, the United States, and Europe concluded that heat-related deaths are preventable if evidence-based alerting and risk communications protocols are present (Benmarhnia et al. 2016).

Prior to the implementation of the HWIS in 2016, there was no consistent approach or terminology for issuing heat warnings in Ontario. PHUs that had systems in place used different triggers for calling heat warnings and public messaging also varied by jurisdiction. Local media would report heat warnings from one PHU that often reached residents in adjacent communities where a heat warning had not been issued, resulting in considerable public confusion. Moreover, the Government of Canada would also issue its own climate-based heat warnings for all regions of Ontario. This further led to situations where a heat warning for a community or region might be called by the federal government but not by the local PHU, or vice versa.

In 2011, PHUs expressed, through workshops and informal discussions, the need to develop a single harmonized heat warning system across Ontario to improve and unify health messaging. This was emphasized in a survey in 2012 where 94% of PHUs surveyed identified as a high or medium priority the need for a more coordinated and consistent methodology for calling heat health alerts. While the 2008 version of the Ontario Public Health Standards required PHUs to increase public awareness of health risk factors associated with extreme weather and climate change (e.g., extreme heat events), those same PHUs were not specifically mandated by the Ontario provincial government to have heat alert and response systems. PHUs across the province had developed a range of independent systems. Several PHUs did not issue heat warnings. However, for those that did, there was often a lack of region-specific health evidence to inform the selection of heat warning triggers. Prior to 2016, many heat warnings were based primarily on climatology and issued by PHUs when extreme daily humidity conditions (humidex) reached 40. It was unclear whether these humidex-based warnings were health protective.

## Intervention

A series of workshops beginning in 2011 brought together partners and generated discussions on how best to collaborate to resolve inconsistencies in existing heat warning systems across Ontario. The partners included public health units, Health Canada, Environment and Climate Change Canada, the Ontario Ministry of Health & Long-Term Care, Public Health Ontario, and Clean Air Partnership, who were retained to facilitate the process. The partners established the Ontario Heat Health Project Team (Project Team) and a Terms of Reference with an objective “to develop an efficient, coordinated, evidence-based system comprised of standardized criteria for calling heat warnings with language easily understood by the public as well as the flexibility to address local vulnerabilities and needs”. See Supplementary Material for a complete list of partners. Working groups were then created to identify tasks and timelines necessary to fulfill the vision and mission.

**Table 1:** Triggers for calling heat warnings in Ontario in the final HWIS. (Source: Ontario Ministry of Health and Long-Term Care, 2016)

Zone (heat warning region)	Condition	Duration
1. Extreme Southwestern Ontario	*T*max ≥ 31 °C and *T*min ≥ 21 °C OR Humidex ≥ 42	2 or more days
2. Southern Ontario	*T*max ≥ 31 °C and *T*min ≥ 20 °C OR Humidex ≥ 40	2 or more days
3. Northern Ontario	*T*max ≥ 29 °C and *T*min ≥ 18 °C OR Humidex ≥ 36	2 or more days

*T*max represents maximum daily temperature. *T*min represents minimum nighttime temperature

### The working groups

In the first year of the collaborative, the Project Team agreed on key priorities and established three working groups:

- Research: Identify knowledge gaps and undertake targeted data analysis.
- Communications: Examine all facets of communication in the context of extreme heat.
- Governance: Identify approaches to address governance issues which affect collaboration around extreme heat and data sharing.

A transparent and inclusive approach to project management ensured positive momentum throughout. For example, decisions of the Project Team were always made on a consensus basis. By 2015, the initial actions identified by the working groups were completed. The Project Team decided to dissolve the original working groups and three new working groups were formed to address outstanding priority issues:

- Alert consistency: Achieve consistency across regions for heat warning triggers, Environment and Climate Change Canada notifications, timing of heat warnings issued by PHUs, terminology, and termination.
- Communications: Develop templates for frequently asked questions, key messages, and media releases.
- Evaluation: Develop a framework to guide evaluation of the harmonized system, including a survey to collect information about the HWIS and the implementation at the PHU level.

### Establishing the health evidence

Health Canada and Public Health Ontario, with input from the Research Working Group, conducted an epidemiological analysis of the impacts of heat on the health of Ontarians to establish evidence-based heat warning triggers for different geographic regions across Ontario (Chen et al. 2016). Additional research included a jurisdictional scan to better understand the state of heat alert and response systems in Ontario, across Canada and internationally. Based on the research, as well as practical considerations around forecast processes, three geographic zones were established in Ontario for calling warnings along with their associated triggers (see Table 1). This approach has ensured that the heat warnings, and the triggers underpinning them, are standardized across Ontario, making it easier to communicate the warnings to the public. At the same time, the creation of the three zones allowed the heat warnings to be tailored to the differences in meteorological conditions and heat health vulnerability identified across the different regions through the epidemiological analysis. These new heat warning triggers were based on Ontario-specific health evidence, replacing the previous patchwork of heat warning triggers, which were often selected without relevant heat health data.

### Identifying consistent terminology

The Project Team agreed upon consistent terminology for communicating heat warnings (i.e., “heat warning”) and for when they extended beyond 2 days (i.e., “extended heat warning”). This was challenging as some PHUs had existing printed communication material using different terminologies that were familiar to local partners. However, there was a strong agreement on the need for consistent language among the partners that helped to overcome this challenge. The partners recognized that consistent language would reduce confusion and increase the likelihood that Ontarians would take appropriate health-protective action.

### Piloting the harmonized heat warning and information system

The Project Team identified the 2015 Pan and Parapan American Games (hereafter, Games) being held in Southern Ontario during the months of July and August as a timely opportunity to test the feasibility of the HWIS. The Ontario Ministry of Health and Long-Term Care, with support from the Governance Working Group, drafted a Standard Operating Practice (SOP) to provide information on the heat warning triggers, terminology, and notification processes. Environment and Climate Change Canada also began issuing heat warnings in accordance with the new triggers across Ontario.

All ten PHUs within the Games’ footprint, and four outside of the footprint, piloted the HWIS for the duration of the Games. A mid-summer check-in with PHUs identified issues and informed adjustments to Environment and Climate Change Canada’s services. The post-heat season survey found that PHUs recognized the value of consistent heat warning terminology. Environment and Climate Change Canada conducted post-heat season interviews with its meteorological forecasters and verified its services, including how it issues early heat notifications as well as the forecast accuracy. Taken together, these post-heat season assessment exercises were important steps for informing refinements to how partners issued heat warnings prior to the launch in 2016.

## Outcomes

**Fig. 1:**
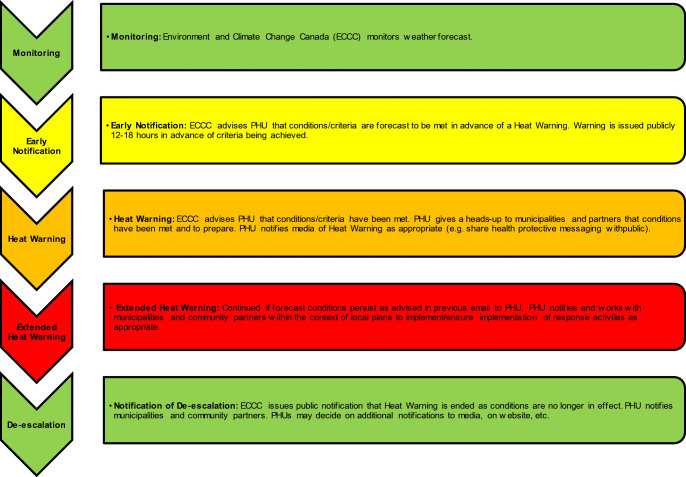
Notification and warning process (Source: Ontario Ministry of Health and Long-Term Care, 2016)

The Ontario HWIS was broadly implemented in 2016 with the Ontario Ministry of Health and Long-Term Care’s release of the harmonized HWIS SOP, which outlined the notification and warning process (Fig. 1). Post-heat season surveys helped the project team measure success in terms of implementation of the harmonized HWIS and experiences reported by PHUs across Ontario. Among all 36 PHUs surveyed, 24 reported that they adopted both the harmonized heat warning triggers and terminology in 2016. This increased in 2017 with 33 PHUs reporting that they had adopted the harmonized heat warning triggers and terminology. Survey respondents provided positive feedback on the HWIS, acknowledging improvement over the previous patchwork of heat warning systems and sharing their heat health activities including outreach to vulnerable populations, monitoring health impacts, and evaluating heat health efforts. These surveys provided a way to measure the success of the harmonized HWIS in that they identified an increase in evidence-based public health practice, in particular the use of Ontario-specific health evidence to establish heat warning triggers.

## Lessons learned

The HWIS project overcame many issues ranging from cross-jurisdictional governance to the varied PHU resources and contexts. The following is a summary of key lessons learned.

### Establish a joint vision early on with all potential partners

All potential partners were engaged at the outset in establishing a shared vision. This early buy-in kept project partners focused on achieving a common goal. Defining this vision made it possible for all partners to agree on common objectives and focus on work areas that were within the scope of the project. Working groups provided regular updates on key deliverables back to the Project Team, which reviewed progress on tasks to ensure the project was on track.

### Allow sufficient time to build consensus

Allowing time to build consensus helped increase trust and understanding between partners. The process took longer than initially expected because of the partners’ varied interests. Time was dedicated at meetings to discuss operational issues, allowing partners to learn from each other and appreciate their respective challenges. While the work leading up to the launch took several years, once there was agreement on the new harmonized system, the implementation rolled out quickly and effectively.

### Leverage and respect the expertise and resources of partners

An asset mapping exercise identified the range of resources that each partner brought to the process, allowing them to take responsibility for implementing actions within their mandates. The participatory approach created the space for PHUs to proactively contribute, take a leadership role, and feel ownership over the initiative. All partners provided important contributions. The PHUs—both those that had experience in issuing heat warnings and those new to the process—provided their expertise in implementing public health protection measures. Health agencies at the provincial and federal levels undertook the analysis of temperature-related health impacts in Ontario that underpinned the heat warning levels. Environment and Climate Change Canada applied their expertise on the meteorological data, in collaboration with PHUs, to operationalize the new heat warning and information system. Respect for the contributions of each partner was key to achieving a harmonized system. Additionally, by coordinating HWIS with multiple levels of government, it opened up a dialogue and opportunities for better collaboration and information sharing during heat events.

### Build an evidence-based system

All Project Team members agreed that the new system should be evidence-based. Public Health Ontario and Health Canada led the analysis of health impacts, while Environment and Climate Change Canada ensured that the weather data and climatology corresponded with the health evidence and could be used in practice by weather forecasters when issuing warnings. The Project Team synthesized the results of the health and meteorological analysis so that each partner could share the information with their respective organizations.

### Be willing to compromise

Some PHUs had existing heat alert systems developed with local stakeholder involvement. Changing these systems meant that materials needed redeveloping and staff had to work with the local community to adjust existing communications and responses. Because trust and a shared vision had been established, PHUs were more willing to compromise, accept, and implement the proposed changes.

### Incorporate feedback throughout the process

Continuous feedback and evaluation was key to ensuring the project met its objectives. Various methods were used throughout the project to ensure that concerns of all partners were addressed. Annual post-season surveys allowed PHUs to learn from each other’s experiences and provided valuable information for provincial/federal partners to inform their services. The long-term success of the HWIS will be assessed through regular feedback from all partners.

### Develop a standard operating practice

The SOP for a harmonized HWIS, released by the Ontario Ministry of Health and Long-Term Care, was a useful tool to ensure clarity and consistency with heat warnings across Ontario. The PHUs were integral to drafting and reviewing the SOP, which helped ensure that the document was relevant to their context.

### Work with a trusted third-party facilitator

An independent facilitator was essential to the success of this initiative. Clean Air Partnership ensured the project maintained momentum and remained on task. As a neutral facilitator, they helped chair meetings to resolve issues and differences of opinion. They were chosen because of their experience working on environmental health issues, their relationship with program leads at all levels of government, and their experience convening multi-stakeholder events. Clean Air Partnership’s flexibility in adapting to the needs of the group was key to their success in facilitating the project and being an intermediary when any issues arose.

### Seize the opportunities when they arise

Timing was key to achieving a harmonized HWIS for Ontario. PHUs were open to changes as some were in the process of assessing their existing heat warning systems, while others lacked a system and were exploring how to develop one. Interest by PHUs coincided with Environment and Climate Change Canada’s interest in strengthening the evidence basis for their heat warnings and Health Canada’s mandate to advance research on heat-related illnesses and deaths across Canada. In addition, the Government of Ontario identified extreme heat as a risk to address at the 2015 Pan Am Games. The Games served as a timely opportunity to pilot a harmonized HWIS.

## Implications and conclusions

As the climate continues to change, many communities across Ontario are expected to experience more frequent and intense extreme heat events. Effectively adapting to and preparing for these events requires a consistent and coordinated health evidence-based approach that is supported by all levels of government. This case study shows how agencies at the local, provincial, and federal level can work together to reach a common objective to better protect health from extreme heat events while allowing each organization to lead components that fall within their mandate. This approach helped create ownership of the initiative among partners as it progressed. The strong interpersonal relationships that developed across organizations were an essential enabling component and also created an atmosphere where people were comfortable speaking openly about challenges and pathways to reach solutions.

The lessons learned from this project have already been useful for spurring action in other regions of Canada. Participation from Alberta Health at a Project Team workshop in 2013 served to catalyze the development of a similar health evidence-based approach to issuing heat warnings in Alberta in 2016. Manitoba and Saskatchewan soon followed using a similar heat warning approach in 2017, and expansion to Atlantic Canada, British Columbia, Yukon, and the Northwest Territories took place in 2018. Meanwhile, the Ontario Ministry of Health and Long-Term Care has continued to move heat warnings forward in the province with the release of their Healthy Environments and Climate Change Guideline in 2018 under which all PHUs are now required to reduce the health impacts of heat events using tools like the HWIS. The development of a harmonized HWIS in Ontario serves as an invaluable model for other provinces and jurisdictions wanting to adopt similar health evidence-based systems.

## Electronic supplementary material

ESM 1 (DOCX 12 kb)

## References

[CR1] Benmarhnia T, Bailey Z, Kaiser D, Auger N, King N, Kaufman JS (2016). A difference-in differences approach to assess the effect of a heat action plan on heat-related mortality, and differences in effectiveness according to sex, age, and socioeconomic status (Montreal, Quebec). Environ Health Perspect.

[CR2] Berry P, Richardson GRA, Steinberg SL, Sprigg W (2016). Approaches for building community resilience to extreme heat. Extreme weather, health, and communities: Interdisciplinary engagement strategies.

[CR3] Bustinza, R., Lebel, G., Gosselin, P., Bélanger, D., & Chebana, F. (2013). Health impacts of the July 2010 heat wave in Québec, Canada. *BMC Public Health, 13*(56). 10.1186/1471-2458-13-56. 10.1186/1471-2458-13-56 PMC3554487 23336593

[CR4] Casati B, Yagouti A, Chaumont D (2013). Regional climate projections of extreme heat events in nine pilot Canadian communities for public health planning. J Appl Meteorol Climatol.

[CR5] Chen H, Wang J, Li Q, Yagouti A, Lavigne E, Foty R, Burnett RT, Villeneuve VJ, Cakmak S, Copes R (2016). Assessment of the effect of cold and hot temperatures on mortality in Ontario, Canada: A population-based study. CMAJ Open.

[CR6] Kosatsky T (2010). Hot day deaths, summer 2009: What happened and how to prevent a recurrence. British Columbia Medical Journal.

[CR7] Lebel, G., Dubé, M., & Bustinza, R. (2019). Surveillance des impacts des vagues de chaleur extrême sur la santé au Québec à l’été 2018. Bulletin d’information en santé environnementale. Institut national de santé publique du Québec. Retrieved from www.inspq.qc.ca/bise/surveillance-des-impacts-des-vagues-de-chaleur-extreme-sur-lasante-au-quebec-l-ete-2018.

[CR8] Lowe D, Ebi KL, Forsberg B (2011). Heatwave early warning systems and adaptation advice to reduce human health consequences of heatwaves. Int J Environ Res Public Health.

[CR9] Ontario Ministry of Health and Long-Term Care. 2016. A Harmonized Heat Warning and Information System for Ontario (HWIS). Toronto, ON: Queen's Printer for Ontario; 2016. Available from: http://www.health.gov.on.ca/en/common/ministry/publications/reports/heat_warning_information_system/heat_warning_information_system.aspx.

[CR10] Pengelly LD, Campbell ME, Cheng CS, Fu C, Gingrich SE, Macfarlane R (2007). Anatomy of heat waves and mortality in Toronto: Lessons for public health protection. Canadian Journal of Public Health / Revue Canadienne de Sante’e Publique.

[CR11] Robine J-M, Cheung SLK, Le Roy S, Van Oyen H, Griffiths C, Michel J-P, Herrmann FR (2008). Death toll exceeded 70,000 in Europe during the summer of 2003. Comptes Rendus Biologies.

[CR12] World Meteorological Organization and World Health Organization. (2015). Heatwaves and health: guidance on warning system development. Retrieved from: http://www.who.int/globalchange/publications/WMO_WHO_Heat_Health_Guidance_2015.pdf.

