# Supplementary material for: Developing a harmonized heat warning and information system for Ontario: a case study in collaboration
Source: Can J Public Health. 2020 Jun 10;111(3):426–32. doi: 10.17269/s41997-020-00337-y (PMC7351991; doi:10.17269/s41997-020-00337-y)
Supplement: Supplementary file 1 — (DOCX 12 kb) [file 41997_2020_337_MOESM1_ESM.docx]

**Supplementary Material**

The authors would like to thank the following individuals who contributed to the collaborative effort through a working group or who contributed through their participation in meetings and requests for input. Note: Affiliations assigned to individuals below are the organizations they represented during the Ontario Heat Health Project Team’s collaboration between 2012 and 2017. The authors recognize that some of these individuals may no longer be employed with these organizations due to retirement or a change of jobs.

Adrienne Hansen-Taugher, Kingston, Frontenac, Lennox & Addington Public Health

Amandeep Hans, Windsor-Essex County Health Unit

Anastasia Rogaeva, Health Canada

Anthony Di Pietro, Durham Region Health Department

Burgess Hawkins, Public Health Sudbury & Districts

Cameron Clarke, Durham Region Public Health

Colleen Farrell, Environment and Climate Change Canada

Diamir de Scally, Health Canada

Elaine Bennett, Windsor Essex County Health Unit

Gabriella Kalapos, Clean Air Partnership

Gagandeep Babra, Peel Public Health

Geoff Coulson, Environment and Climate Change Canada

Haizhen Sun, Environment and Climate Change Canada

Iqbal Kalsi, Middlesex-London Health Unit

Jane Zhang, Toronto Public Health

Jim Frehs, Health Canada

John Cannan, Hastings and Prince Edward Counties Health Unit

Judy Green, Peel Public Health

Kate Bassil, Toronto Public Health

Kiran Ghai, Peel Public Health

Kym Bousfield, Halton Region Health Department

Lori Holmes, Simcoe Muskoka District Health Unit

Marina Whelan, Simcoe Muskoka District Health Unit

Martha Robinson, Ottawa Public Health

Nadia McLennan, Halton Region Health Department

Nena Snyder, Environment and Climate Change Canada

Paul Buttery, Lambton Public Health

Peter Berry, Health Canada

Peter Heywood, Oxford County Public Health

Phil Wong, Algoma Public Health

Rajesh Benny, Toronto Public Health

Rosemarie Arndt, Chatham-Kent Public Health

Sally Radisic, City of Hamilton Public Health Services

Sharon Jeffers, Environment and Climate Change Canada

Stephanie Hamelin, Windsor-Essex County Health Unit

Vidya Anderson, Ontario Ministry of Health and Long-Term Care
